# A Prognostic 14-Gene Expression Signature for Lung Adenocarcinoma: A Study Based on TCGA Data Mining

**DOI:** 10.1155/2020/8847226

**Published:** 2020-12-19

**Authors:** Jie Liu, Shiqiang Hou, Jinyi Wang, Zhengjun Chai, Xuan Hong, Tian Zhao, Zhengliang Sun, Liandi Bai, Hongyan Gao, Jing Gao, Guohan Chen

**Affiliations:** ^1^Department of Thoracic Surgery, Shanghai East Hospital, Tongji University School of Medicine, Shanghai 200120, China; ^2^Research Center for Translational Medicine, Shanghai East Hospital, Tongji University School of Medicine, Shanghai 200120, China; ^3^Department of Cardiology, Zhongshan Hospital, Fudan University, Shanghai 200032, China

## Abstract

**Background:**

Lung adenocarcinoma (LUAD), a major and fatal subtype of lung cancer, caused lots of mortalities and showed different outcomes in prognosis. This study was to assess key genes and to develop a prognostic signature for the patient therapy with LUAD.

**Method:**

RNA expression profile and clinical data from 522 LUAD patients were accessed and downloaded from the Cancer Genome Atlas (TCGA) database. Differentially expressed genes (DEGs) were extracted and analyzed between normal tissues and LUAD samples. Then, a 14-DEG signature was developed and identified for the survival prediction in LUAD patients by means of univariate and multivariate Cox regression analyses. The gene ontology (GO) and Kyoto Encyclopedia of Genes and Genomes (KEGG) pathway enrichment analysis were performed to predict the potential biological functions and pathways of these DEGs.

**Results:**

Twenty-two out of 5924 DEGs in the TCGA dataset were screened and associated with the overall survival (OS) of LUAD patients. 14CID="C008" value=" "DEGs were finally selected and included in our development and validation model by risk score analysis. The ROC analysis indicated that the specificity and sensitivity of this profile signature were high. Further functional enrichment analyses indicated that these DEGs might regulate genes that affect the function of release of sequestered calcium ion into cytosol and pathways that associated with vibrio cholerae infection.

**Conclusion:**

Our study developed a novel 14-DEG signature providing more efficient and persuasive prognostic information beyond conventional clinicopathological factors for survival prediction of LUAD patients.

## 1. Introduction

Lung cancer continues to be the leading cause of cancer-related mortality around the world [1], in which non-small-cell lung cancer (NSCLC) is the most often type, being mainly subdivided into adenocarcinoma (LUAD), squamous cell carcinomas (LUSC), and large cell carcinoma (LCC) [2, 3]. In the past decades, LUAD represents the major lung cancer population, increasingly accounting for approximately 40% of all lung cancers [4]. LUAD were characterized by distinct epidemiological, clinicopathological, and molecular properties [5]. Despite the improvements in diagnosis and therapy made during the past 30 years, the biomarkers for early detecting, prediction of high rate of relapse and mortality populations and the identification of target or immunological therapies for lung cancer patients are still unsatisfactory. Thus, identification of effective biomarkers for the prognosis of LUAD is critical for the diagnosis and treatment of LUAD patients.

Differentially expressed genes (DEGs) that regulated by gene transcription are implicated in diverse biological processes. Gene-expression profiling analysis made some progresses in predicting overall survival (OS) in NSCLC [1, 6, 7]. Mascaux et al. showed that immune activation and immune escape in tumor microenvironment (TME) occurred before lung cancer invasion [7]. With the importance of DEGs involved in cancer research, the roles of DEGs as biomarkers and drivers of tumor oncogenesis and suppression have been identified. However, there are no definite and effective biomarkers in predicting the 5-year survival rate of LUAD patients, which bring great difficulty to clinical prognosis. Therefore, investigation in DEGs may be the solution to noninvasive biomarkers for LUAD.

Although several genes or long noncoding RNA expression signatures, including programmed death-ligand 1(PD-L1), have been recently proposed for predicting the OS in NSCLC [6, 8–10], the prognostic value of an effective and new biomarker of gene profile is still limited. DEG signatures identification related to patient OS in standard clinical samples may promote the development of molecular drug subtypes and potential therapy targets. LUAD and LUSC exhibit distinction in the epidemiology, molecular characteristics, and prognosis [5]. Although several prognostic DEG signatures have been discovered for NSCLC [11, 12], few of these research identify and pinpoint the prognostic value of DEGs biomarkers for LUAD patients in a large cohort. Therefore, we focused on the DEG signature of LUAD not previously published.

In this study, we identified a 14-DEG signature as a predictor of survival risk of LUAD patients using a cohort of 522 cases from The Cancer Genome Atlas (TCGA) database. We employed a survival-associated risk score formula to identify a novel 14-DEG prognostic signature from the TCGA dataset of 522 LUAD patient samples. To show the conscientiousness of this signature, the specificity and sensitivity of our model were examined by the area under ROC curve (AUROC) analysis. A 14-DEG signature which could distinguish patients between good and poor survival was developed by means of Cox regression analysis and risk score model method. A higher area under curve (AUC) of the receiver operating characteristic (ROC) curve confirmed good sensitivity and specificity of the prognostic model, while multivariate Cox regression analysis and stratified analysis indicated the independence of predictive capacity of the 14-DEG prognostic signature. Besides, the functional enrichment analysis demonstrated that the 14-DEG may be probably involved in the progression of LUAD through exerting their roles in LUAD-related function of release of sequestered calcium ion into cytosol and pathways that associated with vibrio cholerae infection. Therefore, our finding may provide insights into the predictive capacity of DEG signature elaborating LUAD.

## 2. Materials and Methods

### 2.1. The LUAD Patient Dataset

The RNA-Seq data set of patients with LUAD was downloaded from the TCGA database (https://cancergenome.nih.gov/), including clinical features. The patients with the following criteria were filtered: patients with complete information of RNA expression profiles and clinical factors (including age, gender, TNM stage, survival status, and survival time).

### 2.2. Differentially Expressed Gene Screening in LUAD

Raw gene-level counts were utilized in our analysis. All the data processing and normalization were performed and completed by using the Perl and R version 4.0.0. The gene expression profiling data of the 522 LUAD samples and 59 normal samples were downloaded from the TCGA database. The DEGs between normal and LUAD group were identified through the “edgeR” package from Bioconductor in R language [13]. ∣log2FC | >2 and adjusted *p* value < 0.01 were set as the threshold for screening the expression difference of DEGs.

### 2.3. Cox Regression Analysis

The RNA-seq expression values were transformed in log2 format to normalize the data. Univariate Cox regression analysis using the “Survival” R package was performed to clarify the association between DEG expression and patient survival. The DEGs (*p* value < 1.0*e*-06) from the univariate analysis were considered as potential candidate DEGs associated with OS. To determine the independent predictive capacity of the 14-DEG signature for LUAD patients, a stepwise multivariate Cox regression analysis was executed to identify the predictive model with the best explanatory and informative efficacy.

### 2.4. Risk Score and ROC Curve

A mathematical formula (Riskscore = ∑_*i*=1_^*N*^(Exp(*i*) · coe(*i*)) ) was developed to predict the risk score for each patient based on the multivariate Cox regression analysis. In accordance with our risk scoring system, patients were classified into high-risk and low-risk groups according to the median risk score. A Kaplan-Meier overall survival curve of the different stages was plotted, and the hazard ratio was calculated. Subsequently, the log-rank test was utilized to determine the survival differences between high-risk and low-risk groups. The sensitivity and specificity of the DEG prognostic model to predict clinical outcome were evaluated by calculating the area under curve (AUC) of the receiver operating characteristic (ROC) curve in the R package of “survival ROC” [14].

### 2.5. Differential Analysis of Scores and DEG Expression with Clinicopathological Stages

The clinicopathological characteristics data corresponding to the LUAD samples were downloaded from TCGA. The independence of the RiskScore from the clinical parameters, such as age, gender, and tumor stage, was determined, and the statistical analysis was performed by Kruskal–Wallis rank sum test or log-rank test as the significance test. In addition, the differential expression of the DEGs between distinct clinicopathologic stages was analyzed and plotted.

### 2.6. Function Enrichment Analysis

Gene ontology (GO) and Kyoto Encyclopedia of Genes and Genomes (KEGG) pathway enrichment analysis was carried out for DEGs with the aid of clusterProfiler R package. Only terms with *p* value < 0.05 were considered as significantly enriched in functions of prognostic DEGs and KEGG pathway analysis.

## 3. Results

### 3.1. Patient Characteristics

**Figure 1:**
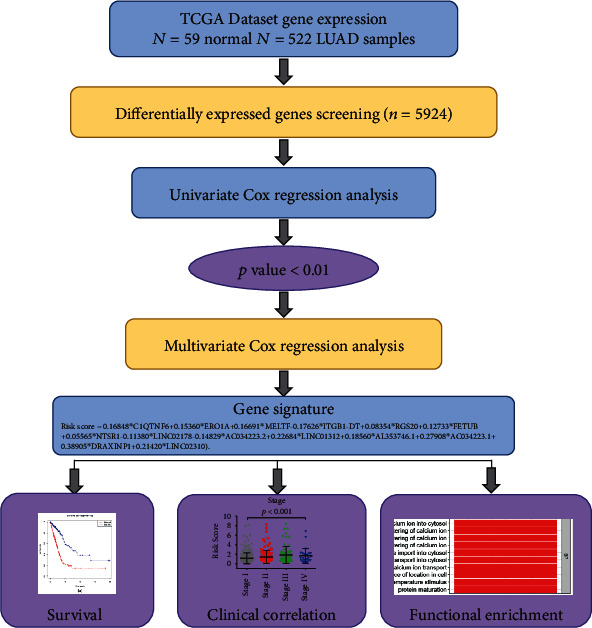
Workflow chart of the gene model construction.

The whole construction process in our research was showed in Figure 1. On basis of the defined criteria, a total of 522 LUAD patients with both RNA-seq expression profiles and clinical data were downloaded from the TCGA. The clinical covariates of the patients in normal or LUAD group were showed in Table 1. 47.89 percent of 522 LUAD patients was no more than and 52.11 percent was more than 65 years old. The female accounted for 53.64% and the male 46.36% in these patients. Of the 522 patients, 280 were classified as stage I, 130 as stage II, while 86 were labeled with stage III and 26 with stage IV disease. The survival time of 522 LUAD patients was 902.51 ± 892.15 days.

### 3.2. Differentially Expressed Genes in LUAD Patients

**Figure 2:**
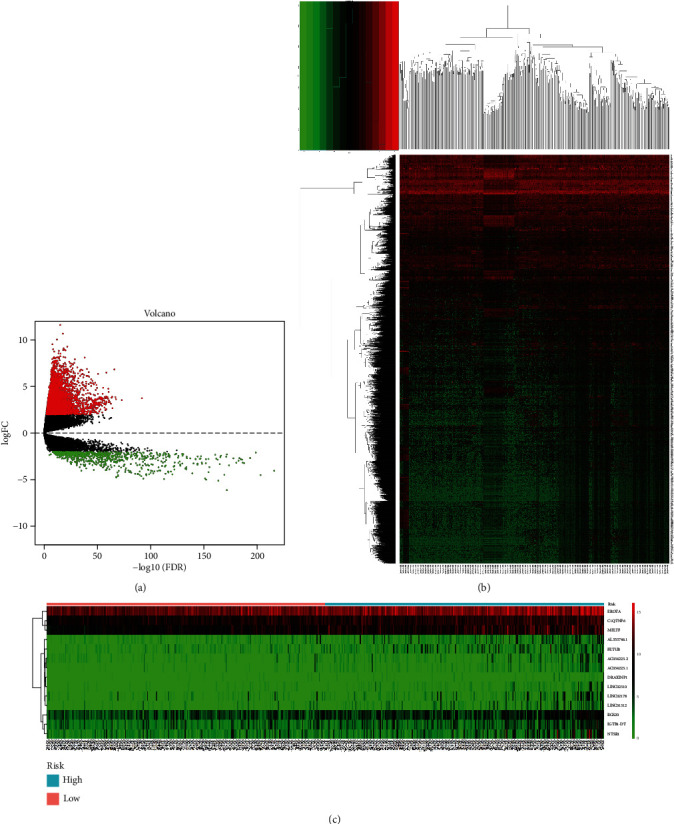
Prognostic evaluation of the 14-DEG signature in LUAD. (a) Volcano plots of DEGs in TCGA dataset. (b) Unsupervised hierarchical clustering analysis of the differentially expressed genes between LUAD and normal tissues. (c) The expression heat map of the 14 DEGs.

According to the defined criteria, a total of 5924 DEGs (including 5147 upregulated and 777 downregulated) were extracted between LUAD and normal samples (Figure 2(a)). The results of unsupervised hierarchical cluster analysis in Figure 2(b) showed that the LUAD samples could be clearly distinguished from the normal controls with the expression of DEGs.

A total of 5924 DEGs were screened to be differentially expressed between LUAD and normal tissues and were used for survival analysis. To identify the DEGs which were related to patient survival in LUAD, univariate Cox regression analysis for all DEG expression data was assessed. With the significance level threshold of 1.0*E*-06, a set of 22 DEGs was selected. These DEGs were utilized in stepwise multivariate Cox regression analysis, and finally, 14 DEGs (C1QTNF6, ERO1A, MELTF, ITGB1-DT, RGS20, FETUB, NTSR1, LINC02178, AC034223.2, LINC01312, AL353746.1, AC034223.1, DRAXINP1, and LINC02310) were identified (Figure 2(c)). The risk score analysis of the 14 DEGs was conducted to calculate the risk score for each patient. The risk score formula for our model was presented in Table 2 (Risk score = 0.16848∗C1QTNF6 + 0.15360∗ERO1A + 0.16691∗MELTF − 0.17626∗ITGB1 − DT + 0.08354∗RGS20 + 0.12733∗FETUB + 0.05565∗NTSR1 − 0.11380∗LINC02178 − 0.14829∗AC034223.2 + 0.22684∗LINC01312 + 0.18560∗AL353746.1 + 0.27908∗AC034223.1 + 0.38905∗DRAXINP1 + 0.21420∗LINC02310). Of these 14 genes, all were associated with high risk (Figure 2(c)).

### 3.3. The Development of the 14-Gene Prognostic Model

**Figure 3:**
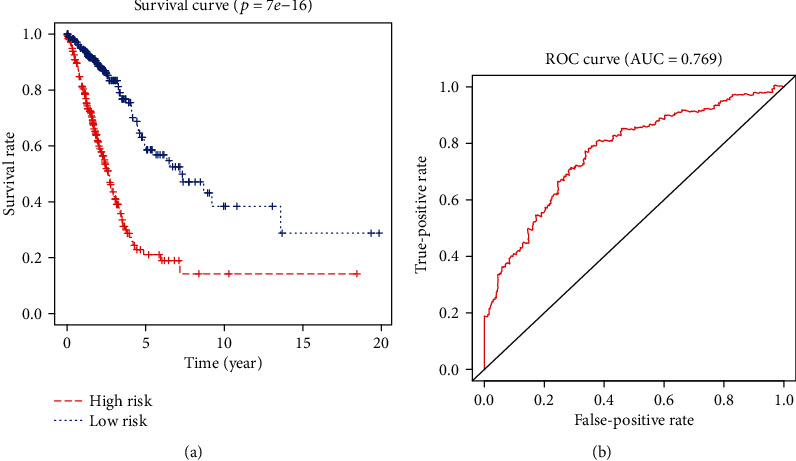
Kaplan-Meier and ROC curves for the 14-DEG signature. (a) Differences between the high-risk (*n* = 252) and low-risk (*n* = 252) groups were determined by the log-rank test (*p* = 7*e* − 16). Five-year overall survival was 21.1% (95% CI: 14.19%-31.4%) and 60.1% (95% CI:50.7%-71.2%) for the high-risk and low-risk groups, respectively. (b) ROC curves indicated that the area under receiver operating characteristic of 14-DEG model was 0.769.

We divided the patients into high-risk and low-risk groups according to the median risk score (value = 0.89) by calculating the expression levels of the 14 DEGs in each patient. The log-rank test was used to determine the survival differences. As depicted in Figure 3(a), Kaplan Meier curves showed that the high-risk group was correlated with poor prognosis (*p* = 7*e* − 16). ROC curves indicated that the AUC of the 14-gene signature was 0.769 (Figure 3(b)), which proved that the 14-gene signature had a high specificity and sensitivity in predicting the OS of LUAD patients.

### 3.4. The 14-DEG Signature Independence from Conventional Clinical Factors

**Figure 4:**
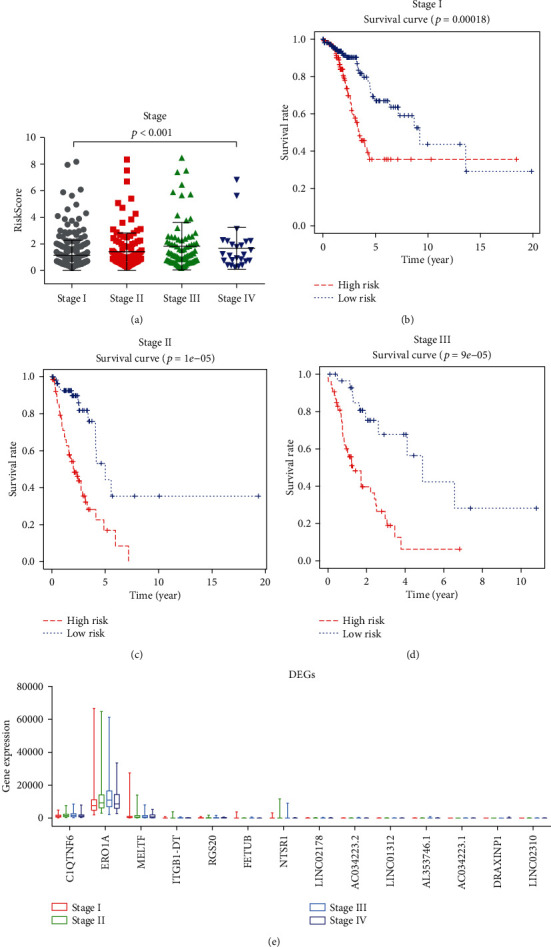
Correlation of RiskScore and survival rate (Kaplan-Meier curves) with clinicopathological staging characteristics. (a) Distribution of RiskScore in TNM stage. The *p* < 0.001 by Kruskal-Wallis rank sum test. (b) Differences between the high-risk (*n* = 116) and low-risk (*n* = 155) groups in stage I patients were determined by the log-rank test (*p* = 2*e* − 4). Five-year overall survival was 35.6% (95% CI: 23.5%-53.8%) and 66.9% (95% CI: 55.8%-80.3%) for the high-risk and low-risk groups, respectively. (c) Differences between the high-risk (*n* = 67) and low-risk (*n* = 57) groups in stage II patients were determined by the log-rank test (*p* = 5*e* − 6). Five-year overall survival was 16.95% (95% CI: 7.09%-40.6%) and 53.1% (95% CI: 33.5%-84.2%) for the high-risk and low-risk groups, respectively. (d) Differences between the high-risk (*n* = 53) and low-risk (*n* = 30) groups in stage III patients were determined by the log-rank test (*p* = 9*e* − 5). Five-year overall survival was 6.31% (95% CI: 1.09%-36.5%) and 42.3% (95% CI: 20.2%-88.6%) for the high-risk and low-risk groups, respectively. (e) Distinct expression of each DEG between different clinicopathological stage samples.

According to multivariate Cox regression analysis, we demonstrated that the 14-DEG signature risk score exhibited an independent predictive ability from other clinical factors (*p* = 7*e* − 16, shown in Figure 3(a)). Meanwhile, we found that TNM stage was an independent factor for predicting the OS of LUAD patients (*p* < 0.001) (Figure 4(a)). Therefore, stratification analysis was further performed to examine whether the 14-gene signature could provide predicted value for patients within the same TNM stage. Because the sample numbers in stage IV were too small to draw any reliable conclusions (*n* = 26), stratification analysis was carried out only in stage I, II, and III patients. Log-rank test for patients in stage I demonstrated that the 14-DEG signature could distinguish patients with significantly different survival time (*p* = 0.00018, Figure 4(b)). Similar predictive outcome of the 14-DEG signature was achieved in stage II (*p* = 1*e* − 05) and III (*p* = 9*e* − 05) patients (Figures 4(c) and 4(d)). Besides, distinct expression of DEGs between different clinicopathological stage samples in Figure 4(e) showed that the DEG expression was positively related to clinicopathological stage. Altogether, these results manifested that the prognostic capability of the 14-DEG signature was independent from conventional clinical factors for predicting survival of LUAD patients.

### 3.5. Functional Enrichment Analysis of Biological Processes and Pathways Correlated with the Prognostic DEGs in LUAD

**Figure 5:**
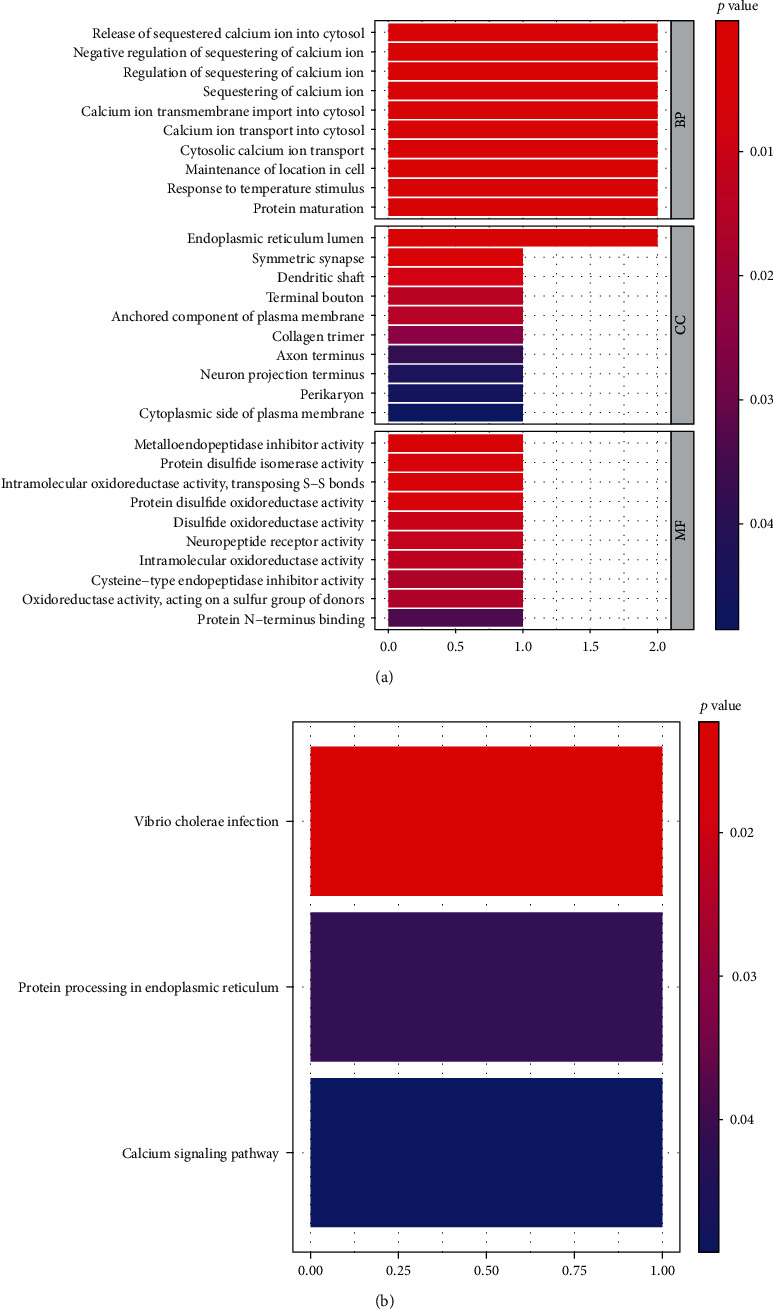
Identification of the DEGs related biological processes and pathways. (a) The functional enrichment analysis of Gene ontology (GO) terms. (b) KEGG pathways for DEGs.

The biological functions and pathway analyses were conducted using R package clusterProfiler. The results showed that DEGs were enriched in 181 GO biological process (BP), 10 cellular component (CC), and 14 molecular function (MF) terms. The GO categories of GO: 0051209~release of sequestered calcium ion into cytosol (BP), GO: 0005788~endoplasmic reticulum lumen (CC) and GO: 0008191~metalloendopeptidase inhibitor activity (MF) were mainly clustered, respectively (Figure 5(a)). The top 10 GO terms were shown in Table 3. The DEGs were enriched in three KEGG pathways which mainly focused on tumor metabolism, including hsa05110: vibrio cholerae infection, hsa04141: protein processing in endoplasmic reticulum, and hsa04020: calcium signaling pathway (Table 4, Figure 5(b)).

## 4. Discussion

NSCLC is a global health threat with high morbidity and mortality, up to 0.6 and 0.1 percent, respectively [11]. LUAD accounts for more than 40% of the lung cancer patients, showing its predominance among NSCLC. On account of the heterogeneity, conventional prognostic systems such as TNM stage sometimes exhibited predicting deficiency for risk stratification and clinical outcome estimations. Therefore, considerable outcomes are in urgent need in recent decades to develop efficient prognostic signatures to promote the prediction of LUAD patient survival.

Increasing evidences suggest that DEGs play indispensable and important roles in the tumorigenesis, TNM staging, and progression of lung cancer. Although several researches have identified a number of DEGs with prognostic value in NSCLC, especially in LUSC [10, 11], few studies have concentrated on and analyzed the DEG expression specifically in LUAD. Moreover, because LUAD and LUSC are vastly distinct diseases at the molecular, pathological classification and clinical level, such as distinct driver genetic changes, response to chemotherapy, or targeted therapy [4, 5], single-gene expression models are insufficient for accurate prediction of LUAD outcomes. Therefore, we focused on the molecular prognostic DEG signature patterns in LUAD.

In this study, 14-DEG signature related to overall survival of LUAD patients was identified. By means of univariate Cox regression analysis and stepwise multivariate Cox regression analysis, a novel 14-gene (C1QTNF6, ERO1A, MELTF, ITGB1-DT, RGS20, FETUB, NTSR1, LINC02178, AC034223.2, LINC01312, AL353746.1, AC034223.1, DRAXINP1, and LINC02310) prognostic signature was established and validated to demonstrate high specificity and sensitivity in predicting the overall survival time of LUAD patients. We calculated the RiskScore of each patient through the formula and the expression of selected DEGs. The patients were divided into high- and low-risk group by the median RiskScore (value = 0.89); then, we obtained the survival curve according to the survival rate of all LUAD patients. To our knowledge, C1QTNF6 has been recently identified as a novel biomarker exacerbating the outcome of lung adenocarcinoma patients [15]. Combined expression of protein disulfide isomerase and endoplasmic reticulum oxidoreductin 1-*α* (ERO1A) is a poor prognostic marker for non-small-cell lung cancer [16]. Level of melanotransferrin (MELTF) in tissue and sera serves as a prognostic marker of gastric cancer. Patients with high serum MELTF levels had poor prognosis [17]. It was demonstrated that ITGA5 and ITGB1 are prognostic in non-small-cell lung cancer by integrin and gene network analysis [18]. Regulator of G protein signaling 20 (RGS20) was identified as molecular marker for LUAD for its effect in enhancing cancer cell aggregation, migration, invasion, and adhesion [19, 20]. Fetuin-B (FETUB) was reported as a plasma biomarker candidate related to the severity of lung function in COPD [21]. Neurotensin (NTS) and its receptor (NTSR1) promote EGFR, HER2, and HER3 overexpression and their autocrine/paracrine activation in LUAD. Their expression is increased in 60% of lung cancer patients. In a previous clinical study, NTSR1 overexpression was applied to predict a poor prognosis for 5-year OS in a stage I lung adenocarcinomas population treated by surgery alone [22]. Besides, LINC02178, LINC01312, AL353746.1, DRAXINP1, and LINC02310 were identified as the prognostic markers and prediction of the survival of LUAD by genome-scale analysis [23]. Among the identified 14 genes in this study, all were associated with high risk, indicating that the expression of these genes was positively related. Moreover, gene MELTF, AC034223.2, and AC034223.1 were firstly identified related to LUAD in our study.

The carcinogenesis of LUAD is a multistep process hallmarked by a series of genetic alterations. In order to gain a further insight into the functional roles of the 14 DEGs, the correlation between their expression levels and the coexpressed protein-coding genes were analyzed. In the present study, we performed GO and KEGG enrichment analysis to explore the functions of the predictive DEGs. The results indicated that the prognostic 14-DEGs were involved in significant functional process, such as release of sequestered calcium ion into cytosol (BP), endoplasmic reticulum lumen (CC), and metalloendopeptidase inhibitor activity (MF) and enriched in KEGG pathways including vibrio cholerae infection, protein processing in endoplasmic reticulum, and calcium signaling pathway. Therefore, it is convincing to infer that the fourteen prognostic DEGs participate in the progression of LUAD in these LUAD-related biological pathways. However, further experimental studies are needed to confirm the functions of these DEGs. Our findings provide insights into prognosis-related genes of LUAD and may have a positive clinical capacity for prognosis prediction and target therapy in LUAD management.

## 5. Conclusions

In summary, this study identified a novel 14-DEG prognostic signature which could predict the survival risk of LUAD patients. The signature exhibited independent prognostic capacity of clinicopathological factors and could predict survival outcomes of LUAD patients within the same TNM stage. This signature could be utilized to identify patients with high-risk scores who may be further desperate for more effective and individualized therapy. It could not only serve as a novel potential biomarker for the survival risk stratification of LUAD patient but also provide us a better understanding of molecular mechanisms involved in the development of LUAD. However, further molecular investigations, such as exploring the underlying mechanisms of these DEGs in LUAD development and performing independent cohorts of large sample sizes from institutions across the country or world, are necessary to confirm accuracy and stability for the prediction signature.

## Figures and Tables

**Table 1:** Summary of LUAD patient clinical characteristics.

Covariates	Group	Patients (*N* = 522)	
		*n*	%
Survival time		902.51 ± 892.15	
Vital status	Alive	334	63.98
	Dead	188	36.02
Stage	I	280	53.64
	II	130	24.90
	III	86	16.48
	IV	26	4.98
T stage	T1	172	32.95
	T2	281	53.83
	T3	47	9.00
	T4	22	4.21
N stage	N0	342	65.52
	N1	99	18.97
	N2	75	14.37
	N3	6	1.15
M stage	M0	496	95.02
	M1	26	4.98
Age	≤65	250	47.89
	>65	272	52.11
Gender	Female	280	53.64
	Male	242	46.36

**Table 2:** 14-DEG risk score model.

DEGs	Coef	Exp (coef)	Se (coef)	*z*	Univariate *p* value	Multivariate *p* value
C1QTNF6	0.168	1.18351	0.0848	1.987	3.230*E*-08	4.694*E*-02
ERO1A	0.154	1.16603	0.10023	1.532	2.970*E*-08	1.254*E*-01
MELTF	0.167	1.18164	0.05823	2.867	6.080*E*-07	4.150*E*-03
ITGB1-DT	-0.176	0.8384	0.06004	-2.936	2.230*E*-07	3.325*E*-03
RGS20	0.084	1.08713	0.0497	1.681	2.030*E*-07	9.280*E*-02
FETUB	0.127	1.13579	0.04674	2.724	5.180*E*-07	6.448*E*-03
NTSR1	0.056	1.05723	0.03601	1.545	9.590*E*-07	1.223*E*-01
LINC02178	-0.114	0.89244	0.06063	-1.877	3.630*E*-09	6.052*E*-02
AC034223.2	-0.148	0.86218	0.09964	-1.488	4.450*E*-07	1.367*E*-01
LINC01312	0.227	1.25463	0.06224	3.645	8.990*E*-09	2.680*E*-04
AL353746.1	0.186	1.20394	0.04763	3.896	1.750*E*-09	9.760*E*-05
AC034223.1	0.279	1.32191	0.11378	2.453	2.580*E*-09	1.417*E*-02
DRAXINP1	0.389	1.47558	0.09525	4.085	1.600*E*-08	4.410*E*-05
LINC02310	0.214	1.23888	0.08501	2.52	1.110*E*-09	1.174*E*-02

Coef: coefficient.

**Table 3:** Enrichment analysis of top 10 GO BP, CC, and MF terms.

ONTOLOGY	ID	Description	*p* value	Count
BP	GO:0051209	Release of sequestered calcium ion into cytosol	0.000383	2
BP	GO:0051283	Negative regulation of sequestering of calcium ion	0.00039	2
BP	GO:0051282	Regulation of sequestering of calcium ion	0.000403	2
BP	GO:0051208	Sequestering of calcium ion	0.000423	2
BP	GO:0097553	Calcium ion transmembrane import into cytosol	0.000539	2
BP	GO:0060402	Calcium ion transport into cytosol	0.000686	2
BP	GO:0060401	Cytosolic calcium ion transport	0.000869	2
BP	GO:0051651	Maintenance of location in cell	0.001275	2
BP	GO:0009266	Response to temperature stimulus	0.001482	2
BP	GO:0051604	Protein maturation	0.002331	2
CC	GO:0005788	Endoplasmic reticulum lumen	0.003556	2
CC	GO:0032280	Symmetric synapse	0.003676	1
CC	GO:0043198	Dendritic shaft	0.011602	1
CC	GO:0043195	Terminal Bouton	0.017058	1
CC	GO:0046658	Anchored component of plasma membrane	0.018268	1
CC	GO:0005581	Collagen trimer	0.026397	1
CC	GO:0043679	Axon terminus	0.039227	1
CC	GO:0044306	Neuron projection terminus	0.044852	1
CC	GO:0043204	Perikaryon	0.046917	1
CC	GO:0009898	Cytoplasmic side of plasma membrane	0.049273	1
MF	GO:0008191	Metalloendopeptidase inhibitor activity	0.00522	1
MF	GO:0003756	Protein disulfide isomerase activity	0.006197	1
MF	GO:0016864	Intramolecular oxidoreductase activity, transposing S-S bonds	0.006197	1
MF	GO:0015035	Protein disulfide oxidoreductase activity	0.007172	1
MF	GO:0015036	Disulfide oxidoreductase activity	0.013332	1
MF	GO:0008188	Neuropeptide receptor activity	0.014624	1
MF	GO:0016860	Intramolecular oxidoreductase activity	0.016238	1
MF	GO:0004869	Cysteine-type endopeptidase inhibitor activity	0.019138	1
MF	GO:0016667	Oxidoreductase activity, acting on a sulfur group of donors	0.019138	1
MF	GO:0047485	Protein N-terminus binding	0.034481	1

**Table 4:** DEG-related KEGG pathways.

ID	Description	*p* value	Count
hsa05110	Vibrio cholerae infection	0.012345	1
hsa04141	Protein processing in endoplasmic reticulum	0.041902	1
hsa04020	Calcium signaling pathway	0.049161	1

## Data Availability

The data used to support the findings of this study are included within the article.
